# Women’s Empowerment in Reproductive Decision-making Needs Attention among Iranian Women

**Published:** 2018-03

**Authors:** Zahra KIANI, Masuomeh SIMBAR, Mahrokh DOLATIAN, Farid ZAYERI

**Affiliations:** 1. Student Research Committee, School of Nursing and Midwifery, Shahid Beheshti University of Medical Sciences, Tehran, Iran; 2. Midwifery and Reproductive Health Research Center, Shahid Beheshti University of Medical Sciences, Tehran, Iran; 3. Dept. of Midwifery and Reproductive Health, School of Nursing and Midwifery, Shahid Beheshti University of Medical Sciences, Tehran, Iran; 4. Dept. of Biostatistics, School of Paramedical Sciences, Shahid Beheshti University of Medical Sciences, Tehran, Iran

## Dear Editor-in-Chief

Women’s empowerment in reproductive decision-making means empowering them in fundamental issues of life (1). The empowerment of women is the foundation of population and development program of countries (2). Women’s health challenges, including deaths due to pregnancy and childbirth, unwanted pregnancy, abortion, and HIV occur in the reproductive period and indicate the inability of women in decision-making and control of fertility. Women’s empowerment leads to health promotion in the community (3).

Health and well-being are closely interrelated and therefore are power. Inability or lack of control in the life is a known risk factor for the disease while the ability is involved in control of life and health. A social impact of women’s empowerment is effect on the health outcomes. Women’s empowerment and their participation has significant positive effects on health and quality of life in the community and Family. Women usually spend money more than men for families’ health, as they are primary care providers in their families. Empowered women tend to take care of themselves and their children (4).

Empowering women is about issues such as the right to decide about the number, timing, and spacing of their children and to decide freely about their reproductive activities (5). In fact, women get a good reproductive health depends on the society’s attention to women’s empowerment. Reproductive health services in a community alone cannot predict the impact of these services on women’s empowerment. Also increase women’s control over fertility and success in family planning is important. In other words, these services must be able to empower women to reach their choices (6). The empowerment upgrades health. The third objective of the Millennium Development Goals emphasis on planning and policy development for empowering women to achieve economic and political opportunities as well as women’s health improvement (7).

Each year more than 222 thousand maternal deaths occur. For every maternal death, at least 30 women suffer from serious diseases and debilitating injuries. Annually 20 million unwanted pregnancies occur and 42 million of these pregnancies ends in abortion (8). Therefore, Cairo Conference Action Plan includes specific steps to increase the autonomy of the women in reproduction with strengthening support of men. Each country must take steps based on their socio-cultural context and so planning several studies is needed to check the status of women in decision making about pregnancy and family planning and the outcomes (7).

Women’s empowerment is difficult to measure because it is: 1) a process; 2) a multi-dimensional concept; and 3) a concept which acts on a different level. Empowerment is usually measured by education, employment and awareness status. Although these factors are good but away from the concept and cannot reflect the empowerment (9). Among the many proposed frameworks for the empowerment, Malhotra and Schuler provided one of the most comprehensive measure for women’s empowerment which is in six different dimensions: economic, socio-cultural, familial, interpersonal, legal, political and psychological dimensions (10).

According to a research, 4 dimensions including cultural, social, family, family planning are proposed for women’s empowerment in reproductive decision making in Iran. An average level of women’s power is shown especially in family planning and social decisions (5, 7).

Therefore, the country requires more attention to this important issue. Women’s empowerment is the third objective of Millennium Development Goals and is a key factor for achieving national development, stability and prosperity of the world’s population are. In fact, women’s access to quality reproductive health depends on a comprehensive attention to women’s empowerment in the context of the community.
